# Mosquito-borne arboviruses of African origin: review of key viruses and vectors

**DOI:** 10.1186/s13071-017-2559-9

**Published:** 2018-01-09

**Authors:** Leo Braack, A. Paulo Gouveia de Almeida, Anthony J. Cornel, Robert Swanepoel, Christiaan de Jager

**Affiliations:** 1 School of Health Systems & Public Health, University of Pretoria, Pretoria, South Africa; 2 Instituto de Higiene e Medicina Tropical, Universidade Nova de Lisboa, Lisbon, Portugal; 3 Department of Medical Virology, University of Pretoria, Pretoria, South Africa; 4 Department of Entomology and Nematology, Mosquito Control Research Laboratory, Kearney Agricultural Center, UC Davis, Parlier, CA USA; 5 Department of Veterinary Tropical Diseases, University of Pretoria, Pretoria, South Africa; 6 Faculty of Health Sciences, University of Pretoria, Pretoria, South Africa

**Keywords:** Africa, Mosquito-borne arboviruses, Vector mosquitoes, Zoonoses, Global health threats

## Abstract

Key aspects of 36 mosquito-borne arboviruses indigenous to Africa are summarized, including lesser or poorly-known viruses which, like Zika, may have the potential to escape current sylvatic cycling to achieve greater geographical distribution and medical importance. Major vectors are indicated as well as reservoir hosts, where known. A series of current and future risk factors is addressed. It is apparent that Africa has been the source of most of the major mosquito-borne viruses of medical importance that currently constitute serious global public health threats, but that there are several other viruses with potential for international challenge. The conclusion reached is that increased human population growth in decades ahead coupled with increased international travel and trade is likely to sustain and increase the threat of further geographical spread of current and new arboviral disease.

## Background

Epidemics in recent years of Zika virus (ZIKV) in South and Central America [1–3] and yellow fever virus (YFV) in Africa [4] and Brazil [5–7] serve as reminders of the dramatic manner in which apparently quiescent or stable zoonoses can flare up or spread with serious international public health, social and economic consequences. These events are steadily increasing in frequency, involving pathogens which establish foothold in new geographical territories where they become endemic after initial rapid spread. Examples include yellow fever introduced to the New World from Africa in the early 1600s with subsequent major impact [8–10], dengue virus which spread as different antigenic variants from its probable Asia-Pacific origin to the Americas in the 1960s and Africa in the 1980s [11–13], chikungunya which arose in Africa to similarly invade extensive new areas in the mid-2000s [14–18], West Nile virus which caused a stir when first diagnosed in the USA in 1999 and soon spread to Canada and Central America [19, 20], and most recently Zika virus with an epidemic in Micronesia in 2007 [21], French Polynesia in 2013 [22] and South America in 2015 [23].

United Nations projections indicate a likely increase in global human population from current > 7 billion people to a probable peak of around 9.6 billion in 2050 [24]. The consequences of such population increase will likely favour the spread and impact of zoonoses. Large human population increases will be associated with increasing population density which facilitates transmission of virus either directly or through vectors. There will also be increased international movement of people by way of migration, tourism or business travel thereby increasing the likelihood of more frequent dissemination of infective sources. Similarly, there will be increased global movement of cargo and trade goods facilitating the spread of vectors, increased land transformation and disruption of historical ecological processes which promotes contact between humans and infected wildlife or sylvatic vectors. Finally, increased human population will also increase breeding site and habitat formation for virus vectors such as *Aedes aegypti* and *Aedes albopictus*, especially in expanding urban environments. A disproportionately high percentage of the mosquito-borne arboviruses currently having serious public health impact at global scale is of African origin. In addition to this subset of arboviruses, there is substantial evidence by way of seroprevalence studies of a wide range of other arboviral infections which - like Zika virus in Africa historically - are circulating within immunologically-adapted indigenous African populations largely having little history of serious symptoms. These include Banzi [25, 26], Bwamba [27–32], Bunyamwera [29, 30, 33–35], Germiston [34, 36], Ilesha [37], Lumbo [38], Middelburg [35], Ndumu [39, 40], Ngari [41–43], Ntaya [44], O’nyong-yong [45], Pongola [29, 30, 35], Rift Valley fever [46, 47], Semliki Forest [35], Shuni [48], Simbu [35], Sindbis [35, 49], Spondweni [50, 51], Uganda S [52], Wesselsbron [29, 30, 34, 35] and Witwatersrand viruses [53]. Given the preponderance of zoonoses of African origin that have escaped previous endemic African settings to make geographical jumps to other regions through anthropogenic processes that are likely to escalate, this paper focuses on the subset of mosquito-borne arboviral zoonoses that are already known to exist in Africa, either with potential for continuing expansion of range or of which very little is known except that they infect humans or have genetic affinities which suggest they may infect humans. When entering immunologically naïve populations in new geographical settings the public health consequences are difficult to predict.

Below we provide an outline of the relevant virus groups and specific viruses that are known to have public health risk or could have public health importance in future, including the mosquito species involved in transmission or found infected with these viruses.

## Synoptic overview of the African mosquito-borne arbovirues of medical importance

Mosquito-borne viruses affecting humans are concentrated in three families, the Flaviviridae (genus *Flavivirus*), Togaviridae (genus *Alphavirus*), and the Bunyaviridae (primarily genus *Orthobunyavirus* but with a few important outliers such as the *Phlebovirus* Rift Valley Fever). For the African region, the individual viruses and their categorization, together with key attributes, are summarized in Table 1. Below follows a summary overview of the virus families and the known African mosquito-borne arboviruses in those families; the arrangement is the same as in Table 1, i.e. alphabetically within family for ease of finding and not clustered into serogroups.

**Table 1:** Summary of key attributes of indigenous African mosquito-borne arboviruses for which some mosquito host data are available

Virus	Acronym	Distribution	Mosquito host species^a^	Known or likely amplifying host	Human infection	Mortalities	Date of first known sustained major geographical jump	Likely mode of rapid, large-distance virus dissemination	Reference
Flaviviridae									
*Flavivirus*									
Bagaza	BAGV	West and Central Africa, India, Spain	*Cx. guiarti*, *Cx. ingrami*, *Cx. perfuscus*, *Cx. thallasius*, *Cx*. *tritaeniorhynchus*, *Cx*. *univittatus*	Unknown	Yes	Partridges and pheasants	Unknown	Migratory birds?	[84, 263]
Banzi	BANV	Angola, Botswana, Mozambique, Namibia, South Africa, Tanzania, Zimbabwe	***Cx. rubinotus***	Rodents?	Yes	Unknown	Unknown	Infected rodents? Overall low likelihood of rapid spread	[25, 62, 64, 264]
Bouboui	BOUV	Central Africa	*Ae. africanus*, *An. paludis*	Unknown	Unknown	Unknown	Unknown	Unknown	[265, 266]
Dengue	DENV1–4	Africa: Angola, Benin, Burkina Faso, Cameroon, Cape Verde, Comoros, Cote d’Ivoire, Djibouti, Democratic Republic of Congo, Egypt, Eritrea, Ethiopia, Equatorial Guinea, Gabon, Ghana, Kenya, Madagascar, Mali, Mauritius, Mozambique, Namibia, Nigeria, Reunion, Rwanda, Senegal, Seychelles, Somalia, South Africa, Sudan, Tanzania, Togo, Uganda, Zambia, Zanzibar; Tropical/sub-tropical Asia-Pacific, north-east Australia; South and Central America	***Ae. aegypti***	Originally non-human primates, now humans	Yes	Humans	From Asia-Pacific to Americas in 1960s and Africa in 1980s	Infected humans and infected *Aedes aegypti*	[11, 13, 69, 75]
Kedougou	KEDV	Central African Republic, Senegal	*Ae. dalzieli*, *Ae. minutus*, *Ae. tarsalis*	Unknown	Unknown	Unknown	Unknown	Unknown	[265–267]
Ntaya	NTAV	Cameroon, Democratic Republic of Congo, Kenya, Nigeria, Uganda, Zambia, Romania	*Culex*?	Unknown	Yes	Unknown	Unknown	Migratory birds?	[82–84, 266]
Spondweni	SPOV	Angola, Botswana, Burkina Faso, Cameroon, Ethiopia, Gabon, Mozambique, Namibia, Nigeria, South Africa	***Ae. circumluteolus***, *Ae. cumminsii*, *Cx. neavei*, *Cx. univitattus*, *Eretmapodites silvestris*, ***Mansonia africana***, ***Mansonia uniformis***	Unknown	Yes	Unknown	Unknown	Unknown	[50, 51, 87, 265, 268]
Uganda S	UGSV	Uganda	*Ae. longipalpis*, *Aedes* spp.	Unknown	Yes	Unknown	Unknown	Unknown	[52, 89, 265]
Usutu	USUV	Africa: Burkina Faso, Central African Republic, Cote d’Ivoire, Kenya, Morocco, Nigeria, Senegal, South Africa, Tunisia, Uganda; Europe: multiple countries	*Ae. albopictus*, *Ae. caspius*, *An. maculipennis*, *Coquellittidia aurites*, ***Cx. neavei***, *Cx. perexiguus*, *Cx*. *perfuscus*, ***Cx. pipiens***, *Cx. univittatus*, *Cx. quinquefasciatus*, *Mansonia africana*	Various bird species (mainly passeriform and strigiform), horses, and possibly also bats	Yes	Frequent epidemic mortalities in Eurasian blackbirds *Turdus merula*	Africa into Western Europe (Spain) in the 1950s, separate strain Africa into Central Europe in the 1970s	Birds via migratory flyways; humans incidental and dead-end hosts	[39, 90–93, 96–98, 107, 269–271]
Wesselsbron	WESV	Botswana, Mozambique, South Africa, Zimbabwe, Zambia, Thailand	***Ae. caballus***, ***Ae. circumluteolus***, *Ae. juppi*, *Ae. mcintoshi*, *Ae. luridus*, *Ae. unidentatus*, *Ae*. (*Neomelaniconian*) spp., *Cx. univittatus*	Livestock	Yes	Sheep	None known, quiescent in southern Africa	Desiccation-tolerant *Aedes* eggs	[34, 35, 55, 62, 100, 101]
West Nile	WNV	Lineage 1: North Africa, Europe, India, Australia, North and South America, Caribbean; Lineage 2: South, Central and East Africa, Madagascar, Europe	*Cx. modestus*, *Cx. neavei*, *Cx. perexiguus*, ***Cx. pipiens***, ***Cx. quinquefasciatus***, ***Cx. univittatus***, ***Cx. theileri***	Birds	Yes	Humans, horses, birds, dogs	1999, North America	Migratory birds; infected humans	[19, 20, 26, 49, 62, 93, 103, 106, 108–110, 114, 116, 122, 123, 128, 264]
Yaounde	YAOV	Cameroon, Ghana	*Cx. nebulosus*	Birds	Unknown	Unknown	Unknown	Birds?	[266, 272]
Yellow fever	YFV	Africa: Angola, Benin, Burkino Faso, Burundi, Cameroon, Central African Republic, Chad, Cote d’Ivoire, Democratic Republic of Congo, Ethiopia, Equatorial Guinea, Gabon, Gambia, Ghana, Guinea-Bissau, Kenya, Liberia, Mali, Mauritania, Niger, Nigeria, Rwanda, Senegal, Sierra Leone, South Sudan, Sudan, Togo, Uganda; Tropical South America, Caribbean	***Ae. aegypti***, ***Ae. africanus***, *Ae. albopictus*, ***Ae. furcifer*/*taylori***, *Ae. leucocelaenus*, ***Ae. luteocephalus***, ***Ae. metallicus***, ***Ae. opok***, ***Ae. simpsoni*** **complex**, ***Ae. vittatus***, ***Haemagogus capricorni***, ***Haemagogus equines***, ***Haemagogus janthinomys***, ***Haemagogus leucocelanus***, ***Haemagogus mesodentatus***, ***Haemagogus spegazzinii***, ***Sabethes chloropterus***	Non-human primates: Africa: Monkeys (*Cercopithecus*), chimpanzees (*Pan* spp.), mangabey (*Cercocebus* spp.), baboons (*Papio* spp.), bush babies (*Galaga* spp.), and possibly hedgehogs (*Erinaceus* spp.); South/Central America: Capuchin (*Cebus* spp), spider (*Ateles* spp.), and howler (*Alouatta* spp.) monkeys and marmosets (*Hapale* spp.)	Yes	Humans	Africa to Caribbean, slave trade prior to 1650	Ship-borne infected *Aedes* eggs or adults; air-transport of infected humans and *Aedes aegypti*	[7–10, 58, 130, 132, 133, 154, 222, 265, 273, 274]
Zika	ZIKV	East, West and southern Africa, Asia Pacific, South and Central America, moving into southern areas of North America	***Ae. aegypti***, ***Ae. africanus***, ***Ae*.** ***albopictus***, *Ae. furcifer*, *Ae. jamoti*, *Ae. opok*, *Ae. flavicollis*, *Ae. grahami*, *Ae. taeniorostris*, *Ae. tarsalis*, *Ae. vittatus*, *Ae. dalziella*, *Ae. fowleri*, *Ae. luteocephalus*, *Ae. metallicus*, *Ae. minimus*, *Ae. neoafricanus*, *An. gambiae*, *Eretmapodites inornatus*, *Eretmapodites quinquevittatus*, *Mansonia uniformis*	Several species of African non-human primates	Yes	Rare in humans	From Asia to Yap Island (Federated States of Micronesia) in 2007, then to French Polynesia and other Pacific Islands 2013 and 2014, and finally to Brazil early 2015	Infected *Aedes* mosquitoes by ship or air; infected humans	[1, 21–23, 52, 75, 139, 140, 144]
Togaviridae									
*Alphavirus*									
Babanki	BBKV	Kenya	***Ae. africanus***, ***Ae. mcintoshi***, ***Ae. ochraceus***, ***Ae. simpsoni***						[93, 265]
Chikungunya	CHIKV	Africa: Benin, Burundi, Cameroon, Central African Republic, Comoros, Democratic Republic of Congo, Equatorial Guinea, Gabon, Guinea, Kenya, Madagascar, Malawi, Mauritius, Mayotte, Mozambique, Nigeria, Republic of Congo, Reunion, Senegal, Seychelles, Sierra Leone, South Africa, Sudan, Tanzania, Uganda, Zimbabwe; Middle East; Europe; Asia; the Americas; Oceania	***Ae. aegypti***, ***Ae. africanus***, ***Ae. albopictus***, ***Ae. cordellieri***, ***Ae. furcifer***, *Ae*. *fulgens*, ***Ae. luteocephalus***, ***Ae. neoafricanus***, ***Ae. taylori***, *Ae*. *vittatus*	Non-human primates, humans	Yes	Humans	Previously limited to Africa and Asia, but since 2004 rapid spread to Europe, the Americas and Oceania with major outbreaks	Infected people; infected mosquitoes in cargo	[14, 17, 62, 75, 151, 152, 155, 156, 158, 159, 275]
Middelburg	MIDV	South Africa, Zimbabwe	***Ae. caballus***, *Mansonia africana*	Ruminants?	Yes	Horses	Unknown	Unknown	[35, 101, 160, 162–164]
Ndumu	NDUV	Southern and East Africa	*Ae. circumluteolus*, ***Ae. mcintoshi***, ***Ae. ochraceus***, ***Ae. tricholabis***, *Cx. rubinotus*, *Mansonia uniformis*	Unknown	Yes	Unknown	Unknown	Unknown	[40, 93, 166]
O’Nyong-Nyong	ONNV	Central, East and West Africa	***An. funestus***, ***An. gambiae***, *Mansonia uniformis*	Unknown	Yes	Unknown	Unknown	Infectious humans moving between areas having appropriate vector species	[45, 151, 167, 169]
Semliki Forest	SFV	Cameroon, Central African Republic, Democratic Republic of Congo, Mozambique, Nigeria, Senegal, Uganda	*Ae. abnormalis*-group, *Ae. argenteopunctatus*	Unknown	Yes	Unknown	Unknown	Unknown	[164, 170, 171]
Sindbis	SINV	Africa: Cameroon, Egypt, Kenya, South Africa, Uganda; Europe and Middle East: Finland, Germany, Israel, Italy, Saudi Arabia, Sweden, UK; East and Australasia: Australia, China, India, Malaysia, Russian Federation, Philippines	***Cx. univittatus***, *Cx. neavei*, *Cx. pipiens*, *Cx. torrentium*, *Culiseta morsitans*, *Coquillettidia fuscopennata*	Migratory and other birds	Yes	Unknown	Entered Europe 1960s or 1970s	Intra or inter-continental via infected migratory birds; infected humans	[62, 93, 106, 110, 115, 172, 175–178, 264]
Bunyaviridae									
*Phlebovirus*									
Rift Valley fever	RVFV	Angola, Botswana, Burkina Faso, Cameroon, Central African Republic, Chad, Congo, Democratic Republic of Congo, Egypt, Gabon, Gambia, Guinea, Kenya, Mali, Mauritania, Mozambique, Namibia, Niger, Nigeria, Senegal, Somalia, South Africa, South Sudan, Sudan, Tanzania, Uganda, Zambia, Zimbabwe, Madagascar, Arabian Peninsula	*Ae. aegypti*, *Ae. caballus*, *Ae. cumminsii*, ***Ae. circumluteolus***, ***Ae. dentatus***, ***Ae. juppi***, ***Ae. mcintoshi***, ***Ae. ochraceus***, *Ae. pembaensis*, *An. squamosus*, *Cx. bitaeniorhyncus*, *Cx. quinquefasciatus*, *Cx. poicilipes*, *Cx. theileri*, *Cx. univittatus*, *Cx. zombaensis*, *Eretmapodites quinquevittatus*, *Mansonia africana*, *Mansonia uniformis*	Cattle, sheep, goats	Yes	Humans, goats, cattle, sheep		Trans-ovarially-infected dessication-resistant *Aedes* eggs	[46, 47, 62, 190–194, 196, 235, 265, 276]
Arumowot	AMTV	Central African Republic, Ethiopia, Ivory Coast, Nigeria, Sudan	*Cx. albiventris*, *Cx. antennatus*, *Cx. rubinotus*	Kurrichane thrush, various mice, gerbils and shrews	Unknown	Unknown	Unknown	Unknown	[277, 278]
*Orthobunyavirus*									
Batai	BATV	Uganda	“Mosquitoes”	Unknown	Unknown	Unknown	Unknown	Unknown	[41]
Bunyamwera	BUNV	Botswana, Cameroon, Central African Republic, Cote d’Ivoire, Guinea, Senegal, Kenya, Namibia, Nigeria, South Africa, Uganda	***Ae. circumluteolus***, ***Ae. mcintoshi***, *An. funestus*	Geese and other water birds	Yes	Unknown	Unknown	Infected humans, desiccation-resistant eggs (but trans-ovarial transmission not proven)	[33–35, 93, 101, 182, 183]
Bwamba	BWAV	Cameroon, Central African Republic, Kenya, Mozambique, Nigeria, South Africa, Tanzania, Uganda	*Ae. furcifer*, *An. coustani*, ***An. funestus***, ***An. gambiae***, *Mansonia uniformis*	Unknown	Yes	Yes	Unknown	Unknown	[28, 31, 32]
Germiston	GERV	Botswana, Namibia, South Africa, Zimbabwe	***Cx. rubinotus***, *Cx. theileri*?	Unknown	Yes	Unknown	Unknown	Unknown	[34, 36, 264]
Ilesha	ILEV	Cameroon, Central African Republic, Ghana, Niger, Nigeria, Senegal, Uganda, Madagascar	*An. gambiae*	Unknown	Yes	Humans	Unknown	Unknown	[37, 184, 185, 279]
Lumbo	LUMV	Mozambique, South Africa	***Ae. pembaensis***	Non-human primates?	Yes	Unknown	Unknown	Unknown	[38]
Ngari	NRIV	Burkina Faso, Central African Republic, Kenya, Madagascar, Mauritania, Senegal, Somalia, Sudan, Tanzania	*Ae. mcintoshi*, *Ae. simpsoni*, *An. funestus*	Unknown	Yes	Possibly	Unknown	Unknown	[41, 43, 93, 280]
Nyando	NDV	Central African Republic, Kenya, Senegal, Uganda	*An. funestus*	Unknown	Yes		Unknown	Unknown	[28, 281, 282]
Pongola	PGAV	Angola, Botswana, Mozambique, Namibia, South Africa, Uganda	*Ae. circumluteolus*, *Ae. mcintoshi*	Unknown	Yes	Unknown	Unknown	Unknown	[28, 93, 268]
Shuni	SHUV	Nigeria, South Africa, Zimbabwe, Israel	***Cx. theileri***	Domestic stock and wild animals?	Yes	Unknown	Unknown	Unknown	[26, 48, 187, 188]
Witwatersrand	WITV	Mozambique, South Africa, Zimbabwe	***Cx. rubinotus***	Birds suspected	Yes	Unknown	Unknown	Unknown	[53, 264]
Rhabdoviridae									
Kamese	KAMV	Central African Republic, Uganda	*Cx. annulioris*, *Cx. pruina*, *Cx. tigripes*	Unknown	Yes	Unknown	Unknown	Unknown	[283, 284]
Mossuril	MOSV	Mozambique, South Africa	***Culex*** **sp**. (*sitiens*?)	Unknown	Yes	Unknown	Unknown	Unknown	[285]

^a^Suspected or known prime vectors in bold

### Flaviviridae

Phylogenetic trees suggest that Africa was the ancestral origin of all mosquito and tick-transmitted flaviviruses [54–56], probably from what were initially non-vectored mammalian viruses [55]. Flavivirus members are readily grouped into distinct clusters, namely mosquito-borne, tick-borne, and a group of non-vectored or no-known vector viruses [55–58]. Their ancient history is tied to the Old World, but in recent times their geographical spread and epidemiologies have been significantly affected by burgeoning human populations and global movements. Tick and mosquito-borne flaviviruses diverged early, with a much more rapid evolution within the mosquito-borne viruses. Ticks change hosts and have blood-meals relatively infrequently whereas mosquitoes have multiple blood-feeds off more hosts in a much shorter time span with greater movement over a landscape given their ability to fly. Mosquito-borne viruses thus have greater opportunity to mutate and spread across shorter generational spans within a greater number of hosts, thereby facilitating evolutionary diversification. Medically-important mosquito-borne flaviviruses can be sub-clustered into two groups. One group comprises viruses associated with *Aedes* mosquitoes several of which cause haemorrhagic disease in primates. The other group subdivided into predominantly *Aedes*-transmitted viruses often also causing haemorrhagic symptoms, and predominantly *Culex*-transmitted viruses often associated with encephalitic disease [55]. Phylogenetic analyses suggest multiple introductions of arboviruses from the Old World to the New World in recent millennia and that the most parsimonious explanation for current genetic distributions indicates an “Out-of-Africa” ancestry; all of the *Culex*-associated flaviviruses currently circulating in Asia, Australia, Europe and the America’s appear to have evolutionary roots in Africa [56]. It has also been posited that, like yellow fever virus, dengue virus similarly had its origin in Africa and was frequently transported to the America’s during the slave period [57, 59]. Other more deeply rooted ancestral lines of viruses dating back 2000 to 3000 years appear to have dispersed eastwards out of Africa and given rise to Japanese encephalitis virus, Murray Valley encephalitis virus and others [56].

#### Banzi virus (BANV)

Banzi virus was first isolated in 1956 from the blood of a febrile child in South Africa [25] and subsequently confirmed from a febrile patient in Tanzania [60]. Seroprevalance studies have shown BANV to be widely distributed across southern Africa including Angola, Botswana, Mozambique, Namibia and South Africa [25, 34]. BANV has been repeatedly isolated from wild-caught *Culex rubinotus* [49, 61] which is regarded as the primary vector of this virus [62]. Rodents are believed to be the natural host [63]. *Culex rubinotus* is widely distributed in Africa and abundant in subtropical coastal marshlands [62]. This mosquito appears to maintain a cycle of viral transfer between rodents but only infrequently feeds on humans [61, 62, 64, 65]. Little else appears to be known regarding BANV.

#### Dengue virus (DENV)

Generally described in the literature as a virus with core distribution in Asia, the historic origin of dengue virus (DENV) remains somewhat elusive and may also have been in West Africa [57]. DENV first appeared in the America’s more or less the same time as yellow fever, implying that DENV and YFV may have been transported on the same slave ships along with the historically African mosquito *Aedes aegypti* [55]. Whatever its historic origins, DENV was first formally described from Japan in 1943 and Hawaii in 1945, epidemics at that time being reported widely across the region from India to the Pacific Islands [13]. From the Asia-Pacific region it spread again to the Americas in the 1970s and Africa in 1984 [13], subsequently dispersing to more than 120 countries mostly in the tropics and subtropics, where it is now one of the most important infectious diseases in the world and causes serious morbidity and mortality [66–68]. DENV has been reported from 34 countries in sub-Saharan Africa [69], fully listed in Table 1. DENV is a complex of four phylogenetically and antigenically distinct serotypes causing dengue fever (DF) and dengue haemorrhagic fever (DHF) in humans. All four serotypes of DENV are believed to have evolved independently from sylvatic origins during the preceding 1 thousand years [70], with strong evidence that DENV was originally a monkey virus [57].

An estimated 50 to 100 million cases of dengue fever and 250 to 500,000 cases of dengue haemorrhagic fever (DHF) are reported throughout the world each year, mostly among young children, with case fatality rates varying from 0.5% to 5% in Asian countries [67, 68, 71–74]. Dengue causes more illness and death than any other arboviral disease acquired by humans [12]. Typical symptoms of DF include high fever, severe headache, myalgia, arthralgia, retro-orbital pain, and maculopapular rash, resulting in incapacitating disease. High fever, bleedings, intense continuous abdominal pain and persistent vomiting are associated with DHF, deteriorating to circulatory failure and altered mental status. Cases of dengue have increased in Africa over the preceding three decades, with major epidemics in West and East Africa in the 1990s and records of dengue in Angola and Mozambique [74, 75]. Dengue virus is transmitted among humans by *Ae. aegypti* and *Ae. albopictus* mosquitoes, the former being the primary vector in urban areas while *Ae. albopictus* is more important in peri-urban and rural environments [68, 72, 76–78]. In addition to the endemic and epidemic cycles in urban and peri-urban areas involving humans as reservoir and virus-amplifying hosts, ecologically distinct sylvatic enzootic cycles are known from West Africa [79] and Southeast Asia, involving non-human primates and sylvatic *Aedes* mosquitoes [80].

#### Ntaya virus (NTAV)

Ntaya virus was first isolated from mice inoculated intracerebrally with a pool of mixed-species mosquitoes collected in Uganda in 1951 [81]. Of the 1318 mosquitoes comprising 20 species in the pool, 1284 were of the genus *Culex*, but the identity of the vector species remains unknown. Antibodies to NTAV indicate presence of the virus in humans from multiple West, Central and East African countries, specifically Nigeria, Cameroon, Central African Republic, Uganda and Kenya [44]. Symptomatic infection manifests with fever, rigors, myalgia, and headache [44]. Serosurveys have revealed antibodies in a variety of migratory birds and domestic animals (1.6% to 13.9% in sheep, cattle, goats, pigs) in Romania [82, 83]. NTAV is neurotropic in birds and causes haemorrhages in the brain, lungs, liver, heart, ovaries, and splenomegaly [84].

#### Spondweni virus (SPOV)

Spondweni virus was first described from virus collected in 1955 from a pool of *Mansonia uniformis* mosquitoes collected in the sub-tropical northern KwaZulu-Natal Province of South Africa [50, 85]. Tests for antibodies among rural people living in the area indicated very low seroprevalence, but two laboratory workers became ill with the virus after working with infected materials. In addition to the initial isolation of SPOV from *M. uniformis*, other mosquitoes hosting the virus subsequently collected from the same area include *Mansonia africana*, *Aedes circumluteolus* and to a lesser degree from *Aedes cumminsi* and *Eretmapodites silvestris* [50]. The majority of isolations from mosquitoes has been from *Aedes circumluteolus* [50, 86]. SPOV infection has been confirmed from countries widely across sub-Saharan Africa (Angola, Botswana, Burkina Faso, Cameroon, Ethiopia, Ghana, Mozambique, Namibia, Nigeria, South Africa), where patients showed symptoms of acute febrile illness including fever, chills, headache, myalgia, arthralgia, nausea, maculopapular and pruritic rash [51, 87, 88].

#### Uganda S virus (UGSV)

Uganda S virus was isolated from a pool of three species of *Aedes* mosquitoes in Uganda in 1947 [52]. High antibody titres were found in 5.8% of 121 human sera from western Uganda and in the serum of one of six wild monkeys. Laboratory infections of rhesus, grivet and red-tail monkeys failed to produce any clinical symptoms and yielded very low titre of circulating virus or complete absence [52]. Another study indicated that under experimental conditions the virus could be maintained in *Ae. aegypti* for up to 79 days and that these mosquitoes appear to be efficient vectors of the virus capable of transmission after an incubation period of less than 10 days [89].

#### Usutu virus (USUV)

Usutu virus was first isolated from mosquitoes in South Africa in 1959 [60]. Recent genetic analysis suggests there are at least six distinct lineages of USUV, the earliest possibly deriving from southern Africa going back to at least the beginning of the sixteenth century [90]. USUV now occurs widely dispersed in Africa reaching from Morocco through to South Africa [91]. USUV has been repeatedly introduced into Europe over the past 50 years, migratory birds using defined flyways being the most likely agent [90]. Using virus phylogenetic relationships and geographical distributions and overlapping this with known migratory bird flyways suggests three primary routes whereby USUV likely spread into and across Europe: an east Atlantic pathway linking Africa with western Europe (Spain), a Black Sea/Mediterranean pathway to central Europe, and an East Africa/West Asian pathway. The current range of virus distribution in Europe suggests an initial introduction into western Europe (Spain) in the 1950s, followed by introduction of a separate strain into central Europe in the 1970s [90]. The virus causes large-scale die-off among Eurasian blackbirds (*Turdus merula*) in Europe, but USUV has also been found in at least 29 other species of birds covering several families and including species known to undertake long-range inter-continental migrations such as storks, kestrels and swallows [92]. Human infections have been diagnosed from Africa and Europe, with symptoms including fever and rash but sometimes also jaundice and severe neurological impairments [93–95]. USUV has been isolated from numerous mosquito species but vector competence remains poorly understood [96]. The 1959 isolation of USUV in South Africa was from *Culex neavei* [60], with subsequent extractions from *Culex pipiens* in Kenya [93], *Aedes albopictus*, *Aedes caspius*, *Culex perexiguus* and *Cx. pipiens* in Europe [96], and *Culex perfuscus*, *Coquellittidia aurites* and *Mansonia africana* in Senegal [97]. *Culex neavei*, from which numerous isolations of USUV have been made, occurs widespread across Africa and has strong ornithophilic feeding behaviour with high vector competence indices and is abundant at tree-top level where birds rest, which suggests it is an important vector in Africa [96]. In Europe *Cx. pipiens* is considered to be the most important vector [92]. It appears that the virus is transmitted and maintained in a sylvatic cycle that involves mainly *Culex* mosquitoes and birds as the main amplifying hosts, humans being occasional incidental and dead-end hosts; USUV has also been found in bats in Germany [90, 98].

#### Wesselsbron virus (WESV)

Wesselsbron virus was first isolated in 1955 as the aetiological agent responsible for abortion and high mortality among sheep during outbreaks in South Africa [99]. The virus or antibodies to it was subsequently found to be common in humans across much of the tropical eastern coastal lowland of Mozambique, Botswana [34] and northern South Africa [35, 62] as well as in cattle, sheep and goats across the same region and extending into Zimbabwe and Zambia [62, 100, 101]. Gould et al. [55] indicate that WESV is also present in Thailand. Various *Aedes* species have been confirmed to be involved in epizootic transmission of the virus, namely *Aedes caballus*, *Ae. juppi*, *Ae. mcintoshi*, *Ae. luridus* and *Ae. unidentatus* on the temperate South African Highveld plateau while *Ae. circumluteolus* is likely the main vector in the low-lying coastal areas of northern South Africa and in Mozambique. These mosquitoes are floodwater-breeding species, with eggs adapted to survive long dry spells, and the possibility therefore exists that vertical transovarial transmission of virus may occur between successive generations of mosquitoes [62]. Other wild-caught mosquitoes found to be infected with WESV include *Mansonia uniformis*, *Culex univittatus* and pools of mixed *Aedes* (*Neomelaniconian*) species [100].

#### West Nile virus (WNV)

West Nile virus was isolated and described from blood of a febrile patient in Uganda in 1937 [102]. The disease occurs widespread throughout virtually all countries in Africa from Egypt to South Africa, and also the Middle East, southern Europe, Asia, Australia and the Caribbean, North, Central and South America [20, 103–106]. Phylogenetic trees constructed from a wide range of WNV isolates suggest that WNV originated in Africa and dispersed into Mediterranean countries and Europe, from there radiating out to its current distribution [55]. It is now considered the most important causative agent of viral encephalitis worldwide [104]. Infrequent outbreaks of mild febrile disease have occurred in France, India, Israel, Egypt and South Africa since the 1950s but the frequency, severity and geographical range has increased since the mid-1990s [104]. The scale of outbreaks can be very large, as demonstrated by an outbreak in 1974 which involved tens of thousands of human cases over an area of approximately 2500 km^2^ in the Karoo region and Northern Cape Province of South Africa. Following the outbreak, mean antibody prevalence averaged 55% among humans but rose to 85% in some locations, while antibodies were detected in 53% of wild birds examined [26, 61]. WNV was accidentally introduced into New York in 1999 where it caused 62 cases of human encephalitis and 7 deaths. It subsequently spread across the continental USA, Canada, Mexico, Colombia and the Caribbean [19, 105]. In the USA, where WNV is now endemic, a cumulative total of 17,463 cases of neuroinvasive disease and 1668 fatalities were attributed to WNV between 1999 and 2013 [107].

Multiple genetic lineages of WNV are recognized, of which lineages 1, 2 and 5 have been associated with outbreaks in humans [104]. Lineage 1 is the dominant form and occurs in North Africa, the Middle East, Europe, India, the Americas and Australia [20, 103, 104, 108, 109]. Two sub-lineages are recognized, most within one sub-lineage and the other often referred to as Kunjin virus which has only been found in Australia [104, 108]. Lineage 2 was previously considered to be a purely Central/Southern Africa and Madagascan zoonosis [108, 110, 111], but since 2004 has been confirmed at several sites in Europe [20, 111, 112]. Lineage 5 was designated as a distinct separate form associated with 13 human cases in India occurring between 1955 and 1982 [113]. In humans, most WNV infections are asymptomatic but often manifest as febrile illness with headache, rash, fatigue, myalgia and arthralgia; severe infections may lead to paralysis, seizures or cerebellar ataxia with associated long-term cognitive and neurological impairment [107, 114], and a mortality rate close to 10% among patients with neuroinvasive disease [19]. Human fatalities are mostly associated with young children and elderly patients [115]. Other vertebrates are also susceptible: a large epizootic of WNV encephalitis occurred among equines in the USA in 2002, with 14,571 cases reported and a case-fatality rate approaching 30% [105]. In southern Africa, WNV is frequently diagnosed among horses, often causing neurologic disease and sometimes fatalities [106, 109, 116]. Similar WNV infection and deaths have been reported among horses in Italy [117] as well as horses and donkeys in France with up to 34% mortality among those displaying neurologic symptoms [118]. WNV-associated mortality - sometimes very high - has also frequently been recorded among bird populations in multiple countries [19, 105, 107, 119–122]. WNV uses a wide range of bird species as amplifying hosts and is transmitted by various *Culex* mosquitoes, including the widespread *Cx. pipiens* and *Cx. quinquefasciatus* [108, 123, 124]. In southern Africa the principle vector is *Cx. univittatus*, with *Cx. theileri* as a minor vector. In Europe the principal vector is *Cx. pipiens* [20, 108, 125], while in the USA the three species *Cx. pipiens*, *Cx quinquefasciatus* and *Cx. tarsalis* are primary vectors dominating in different parts of the mainland USA [104, 126, 127]. Because viraemia is typically low in humans and equines, the mosquitoes get their infections from birds from where it is passed on to humans in subsequent blood-feeding. For this reason human and equine epidemics of WNV are usually associated with concurrent bird epizootics [62, 128].

#### Yellow fever virus (YFV)

Yellow fever virus originated in Africa and spread with the slave trade dating back to at least 1650 [8, 58, 129]. Barbados in the Caribbean was uninhabited in the early 1600s but then heavily planted with sugarcane to satisfy burgeoning European demand. Large numbers of slaves were imported from Africa along with *Ae. aegypti* and YFV-infected people, subsequently infecting mainly non-immune white overseers on the islands. Major outbreaks of YF occurred from 1647 to 1650 and again in the 1690s, driving many whites to North America where they established a new focus for YF to spread more widely across the Caribbean and South America. The impact of YFV was manifested most heavily during construction of the Panama Canal, which began in 1881. Soon two out of every three Europeans working on the project died from either yellow fever or malaria and ultimately approximately 30,000 would die before the Canal initiative collapsed. Following the Spanish-American War in 1898, approximately 80% of American occupying forces in Cuba contracted yellow fever. The American Congress established a Commission headed by Walter Reed to investigate YF and in 1900 the link with *Ae. aegypti* was established and measures for mosquito control implemented. America took over the half-completed Panama Canal and finished the project in a decade, with approximately 2% of the workforce in hospital at any given time due to a combination of yellow fever and malaria [10]. YF continues to cause periodic outbreaks in Africa where it is known from 28 countries (Table 1). Epidemics can be extensive such as in Ethiopia between 1960 and 1962 estimated to have involved around 100,000 cases with 30,000 deaths, and another surge between 1984 and 1990 during which 22,647 cases were reported from Africa, of which 21,299 were from Nigeria [130]. A recent outbreak involving all 18 provinces in Angola commenced in December 2015 and by July 2016 had resulted in more than 3552 cases with 355 mortalities [4, 131]. Typically only about 15% of persons infected with YFV develop clinical symptoms and the majority of those have mild disease and recover quickly. Usual presentations include sudden onset of fever, headache, muscle pain, backache, general weakness, red eyes, nausea and vomiting, lasting 2–4 days usually followed by uneventful recovery. However, severe disease can develop following a brief period of remission of symptoms lasting up to 24 h after which a second “toxic” phase sets in associated with high fever, vomiting, epigastric pains, jaundice, haemorrhagic diathesis (hematemesis), coma and death [129, 130]. Under African sylvatic conditions YFV is transmitted primarily by *Aedes africanus*, *Ae. furcifer/taylori*, *Ae. luteocephalus*, *Ae. metallicus, Ae. vittatus* and *Ae. opok* [58, 130, 132, 133]. In South America a sylvatic cycle has also been established involving several *Haemagogus*, *Aedes* and *Sabethes* mosquitoes [58]. This sylvatic source is now the primary form of YFV infection in South America, and the disease has had no significant urban transmission in recent decades [8]. The sylvatic reservoir hosts in Africa are a range of primates including monkeys of the genus *Cercopithecus*, chimpanzees (*Pan* spp.), mangabey (*Cercocebus* spp.), baboons (*Papio* spp.), bush babies (*Galaga* spp.), and possibly hedgehogs (*Erinaceus* spp.), while in South and Central America the reservoir hosts involved are capuchin (*Cebus* spp.), spider (*Ateles* spp.), and howler (*Alouatta* spp.) monkeys and marmosets (*Hapale* spp.). Infected forest primates have a period of viremia lasting 1–6 days during which time they may transmit virus to mosquitoes, presumably then developing lifelong immunity. All African non-human primates except bush babies appear to be relatively tolerant to YF virus infection, in most cases developing mild symptoms but rarely leading to fatal disease. In Central and South American monkeys and marmosets, fatal infections are the rule and extermination of focal primate populations rather than development of an immune population commonly accounts for the termination of epizootics. Experimental studies have shown that Asian monkeys are very susceptible to YF virus infection, commonly developing acute and fatal disease [9]. Yellow fever is now overwhelmingly transmitted between humans by *Ae. aegypti* as a domestic/peri-domestic disease [11]. Generally, more than 90% of cases occur in sub-Saharan Africa [134], where an estimated 51,000 to 380,000 severe cases of yellow fever occur each year with 19,000 to 180,000 deaths [67, 135]. Given the widespread distribution and abundance of *Ae. aegypti* in Southeast Asia, with high human population densities ideal for disease spread, it remains a perplexing anomaly why yellow fever has not made the jump to that region [136–138]. China has an increasingly strong expatriate community in Africa, including Angola from where a number of returning workers have been diagnosed with imported YFV in various parts of China, but thus far have not resulted in establishment of local infectious foci and local transmission [138].

#### Zika virus (ZIKV)

Zika virus was first described from a sentinel rhesus monkey placed in the Zika Forest in Uganda in 1947, and from *Aedes africanus* mosquitoes collected in the same forest in 1948 [139]. Subsequent serological and other studies showed ZIKV to be widespread across the eastern and western parts of Africa, with a relatively low seroprevalence of 0.5% in French Equatorial Africa in 1954 but a much higher prevalence of 38% in a 1971–1975 survey in Nigeria [140]. Other studies indicate that ZIKV has been circulating widely over Africa for decades, with exposure to the virus in at least 25 countries across the continent [141]. Until recently southern Africa south of the Zambezi River was considered to be free of Zika [142, 143]. However, an overlooked publication in Portuguese [30] reported on serosurveys showing that 10 of 249 (4%) persons sampled at 22 localities along the length of Mozambique tested positive for Zika neutralizing antibodies [29]. Genetic investigations revealed a distinct strain of ZIKV which has been circulating across much of Asia since at least 1951, including India (1952), Thailand (1954), the Phillippines (1953) and Indonesia (1951); it is suspected that this lineage of ZIKV was probably already present in Asia during World War 2 but misdiagnosed as the far more common dengue, there being extensive cross-reactivity between Zika antibodies and dengue virus [21, 23]. ZIKV remained endemic in Africa and Asia for decades, until the Asian ZIKV lineage caused an epidemic on Yap Island (Federated States of Micronesia) in 2007 [21], and from there jumped to French Polynesia in late 2013 [22], spreading to a series of Pacific Islands between 2014 to 2016 [1] and to Brazil in early 2015 [3, 23], now advancing across South America and into Central America [1]. The average rate of spread of Zika within Brazil after initial introduction has been calculated as approximating 42.1 km/day [3]. Three distinct genotypes of ZIKV are recognized: West African (Nigerian cluster), East African (MR766 prototype cluster) and Asian. It has been postulated that ZIKV originated in East Africa from where it spread to West Africa and Asia [144]. The majority of ZIKV infections are asymptomatic [87]. Clinical symptoms of ZIKV illness are typical of many other arboviral infections such as chikungunya and dengue, and include fever (usually mild), rash, arthralgia, arthritis, myalgia, headache, conjunctivitis, and edema; severe cases requiring hospitalization are uncommon and fatalities rare [87, 140]. However, it is the consequences of infection in pregnant women many of whom give birth to babies with a range of eye, cardiac and neurological anomalies such as microcephaly that is the major concern [1]. ZIKV infections have also been linked to Guillain-Barré syndrome in at least seven South American countries [145, 146]. Historically ZIKV was likely to have been maintained in Africa as a sylvatic cycle involving non-human primates and *Aedes* mosquitoes, with humans as incidental hosts. Elsewhere however, sylvatic cycles appear not to exist but transmission flourishes as a human-mosquito-human cycle in impoverished urban settings, involving *Ae. aegypti* and to a lesser extent also *Ae. albopictus* as primary vectors [1]. ZIKV has been isolated from a wide range of mosquitoes, including at least 17 species of *Aedes*, the malaria vector *Anopheles gambiae*, two species of *Eretmapodites*, and *Mansonia uniformis* [21]. Despite some earlier conflicting reports, it has nevertheless been shown that *Culex quinquefasciatus*, and possibly other *Culex* species, are not effective vectors [147–149] and also *An. gambiae* and *Anopheles stephensi* [147].

Additional flaviviruses for which some mosquito host data could be found are listed and basic data provided in Table 1, but not outlined here as very little is known about them; these include Bagaza, Bouboui, Kedougou and Yaounde viruses.

### Togaviridae

The genus *Alphavirus* of the Togaviridae comprises a large group of medically-important mosquito-borne viruses somewhat complex in the origin, dispersal and ecology of member species. Current phylogenetic tree analyses and supporting indicators suggest that alphaviruses may have arisen from an ancestral aquatic habitat, as reflected in the Southern elephant seal virus SESV and various fish viruses, a poorly studied field. Spread then expanded from southern oceans to both the New World and Old World, invading terrestrial hosts, with historical repeated re-introductions across the globe pre-dating significant-scale human shipping and other transport likelihood. Zoonotic hosts are likely to have assisted this spread, in particular birds, but the picture and its underlying evidence requires further study [150]. Unlike flaviviruses which have clear evolutionary and ecological relationships with specific vector groups, the alphaviruses are less clustered. *Aedes* and *Culex* mosquitoes are important vectors, but with other genera such as *Anopheles* also involved. Alphaviruses have subgenomic promoters and dual polyproteins that facilitate more frequent changes in host range and vector range, plus a capacity for more mutations to be incorporated [150]. This has epidemiological consequences that make for more rapid spread of alphaviruses and also genetic recombination.

#### Chikungunya virus (CHIKV)

The first accurately diagnosed outbreak of Chikungunya (CHIKV) was described from an epidemic in Tanzania in 1952 [17]. Multiple subsequent outbreaks occurred in Africa and SE Asia in the period after 1953 [14, 151]. However, a surge of spread and large outbreaks from the mid-2000s initiated concern, associated with a mutation within CHIKV, enabling it to more easily multiply within the midgut of vector mosquitoes and leading to a 100-fold increase in viraemia within the salivary glands [14, 152, 153]. Three genotypes are recognized: East/Central/South African, West African, and Asian [15]. Commencing in 2004, near global explosive epidemics occurred originating from the East/Central/South African lineage, spreading to Indian Ocean islands, India, Asia, and also Europe and the Americas, where it affected millions of people. An outbreak in the Indian Ocean island of La Reunion in 2005–2006 infected 266,000 people, approximately one-third of the human population. In India in 2006–2007 an epidemic of CHIKV resulted in 1.42 million reported cases [16]. Thailand reported in excess of 49,000 cases for 2008–2009 [154]. CHIKV had not been known from the Americas prior to 2013, but the presence and autochthonous transmission of the virus was reported from the Caribbean island of St Martin in December 2013, and then very rapidly spread within the next year to be reported from 43 countries or territories in the Americas with more than 1.1 million suspected cases. During the period January 2015 to November 2016 an adiitional 1.2 million cases were reported from the region [154]. CHIKV is now widespread across the globe, giving rise to increasing concern as a public health threat. Phylogenetic studies indicate that the eastern half of Africa is the historic origin of CHIKV from where it subsequently spread; CHIKV is closely related to two other arboviruses of African origin, o’nyong-nyong and Semliki Forest viruses [17, 18]. In Africa, local transmission of CHIKV has been reported from most sub-Saharan countries [155], with a full list of affected countries listed in Table 1. Symptoms of CHIKV include fever, rash, arthralgia, myalgia and headache, and may in rare instances lead to severe manifestations of neurological disease, myocarditis, and multi-organ failure, which may be fatal [156]. Infection is usually not life-threatening, but can persist for years associated with pain and swelling mostly in the wrists, hands, ankles and feet [157]. Important sylvatic vectors in Africa are *Aedes africanus*, *Ae. furcifer*, *Ae. cordellieri* [62, 158], also *Ae. taylori*, *Ae. neoafricanus* and *Ae. luteocephalus* [151], but under urban conditions *Ae. aegypti* and *Ae. albopictus* are the main vectors [156]. *Aedes albopictus* is acknowledged as the most important global vector of CHIKV [159], in large part due to a genetic mutation within the virus which enabled it to increase transmissibility to *Ae. albopictus* but with little impact on infectivity to *Ae. aegypti* [17]. Historically, CHIKV was maintained in an enzootic sylvatic cycle in Africa involving wild primates and forest-dwelling *Aedes* mosquitoes, with an abundance of serological presence in wild primates and humans throughout the moist forests and semi-arid savannas of Africa [18, 151]. Since the spread of CHIKV from Africa to other geographical regions, endemic/epidemic transmission cycles have been established with *Ae. aegypti* and *Ae. albopictus* mosquitoes transmitting the virus to humans in a mainly urban human-mosquito-human cycle [14].

#### Middelburg virus (MIDV)

Middelburg virus was isolated in 1957 from pools of *Aedes* mosquitoes during an outbreak of disease among sheep in the Middelburg area of the Cape Province in South Africa [160]. Subsequent antibody surveys in what is now the KwaZulu-Natal Province of South Africa showed positive reactions for MIDV in humans, cattle, sheep and goats [35, 101]. Tissue samples from 623 horses with unexplained febrile and acute neurologic infections sent in by veterinarians across South Africa for diagnostic assays between 2008 and 2013 indicated 44 (7.1%) positive for MIDV, several co-infected with other viruses such as SHUV and WNV [161]. In horses, symptoms of infection can be severe and resemble that of African Horse Sickness [162], including fever, stiffness, swollen limbs, hyperreactiveness, and depression, frequently associated with neurological manifestations such as paralysis, recumbency and seizures, occasionally ending in death [161, 163]. Specific mosquito species that have been found to host the virus include *Aedes caballus* and *Mansonia africana* [164]. Aside from South Africa, MIDV has also been recorded from Zimbabwe, Cameroon, Kenya, Senegal and the Central African Republic [164, 165].

#### Ndumu virus (NDUV)

Ndumu virus was first described in 1961 from pools of mosquitoes captured in the northern KwaZulu-Natal area of the eastern seaboard of South Africa [40]. Intracerebral and intraperitoneal injection into new-born and adult mice was fatal with lesions typical of viral encephalitis. A non-immune vervet monkey *Cercopithecus aethiops pygerythrus* inoculated intracerebrally developed viraemia but no signs of illness. Serological tests on rural people in eight widely scattered locations suggest that NDUV is endemic over a large area of southern Africa but no known associated morbidity [39, 40]. The mosquitoes from which virus was obtained in South Africa were *Mansonia uniformis* and *Aedes circumluteolus* [40]. In Kenya, a survey at three different habitat locations during which a total of 450,680 mosquitoes were captured, revealed that most mosquitoes and most virus was present at a floodwater location and especially from the floodwater-breeding *Aedes mcintoshi, Aedes ochraceus* and *Aedes tricholabis*, which yielded high levels of NDUV but also other viruses such as Sindbis, Babanki, Usutu, Bunyamwera, Pongola and Ngari; lakes and other large bodies of water were dominated by other genera of mosquitoes and some NDUV isolates were obtained from *Culex rubinotus* and *Mansonia* species [93, 166].

#### O’nyong-nyong virus (ONNV)

O’nyong-nyong virus was isolated as the cause of a major epidemic of dengue-like illness in 1959–1962 involving approximately 2 million people, commencing in north-west Uganda and spreading to adjoining Congo, Sudan, Kenya and Tanzania, ultimately reaching Mozambique to the south and Senegal to the north-west [45, 167]. During 1996–1997 a second epidemic of ONNF occurred, restricted to south-central Uganda but with high infection rates of between 45 and 68% of people in affected regions; symptoms of the disease included acute fever with associated polyarthralgia but no known fatalities [45]. Initially suspected of being a new and separate virus, the closely-related *Igbo-Ora virus* which occurs in west-central Africa manifests with very similar symptoms; genetic analyses suggest it is a strain of ONNV [168]. ONNV has been repeatedly isolated from *Anopheles funestus* and *Anopheles gambiae* in different areas, and the fact that ONNV is absent from one particular area in Uganda from which the two mosquito species are also absent but both occur in neighbouring regions provides strong evidence that the main vectors are indeed these two anthropophilic mosquito species [167]. ONNV is unique among the mosquito-borne viruses in that it is the only known virus with Anopheline mosquitoes as primary vectors, unlike other viruses that have primarily Culicine vectors [151]. However, ONNV has also been found in one pool of *Mansonia uniformis*, a common and widespread mosquito in Africa which also readily feeds on humans, thus demonstrating the potential for this species to play a role in ONNV epidemiology [169]. A vertebrate reservoir host for ONNV is not known [151].

#### Semliki Forest virus (SFV)

Semliki Forest virus was first isolated from *Aedes abnormalis*-group mosquitoes captured in 1942 in Uganda [170]. SFV was also isolated from *Aedes argenteopunctatus* mosquitoes collected in central Mozambique in 1959 [171]. Mice injected with SFV invariably developed hind-leg paralysis and convulsions followed by death [170, 171], while rabbits showed fever and paralysis [170]. Rhesus monkeys injected with SFV did not show any subsequent symptoms [170] but infected vervet monkeys showed fever with no other symptoms [171]. Little appears to be known regarding clinical symptoms in humans, although serosurveys from rural inhabitants in the coastal lowlands of northern South Africa and in central Mozambique revealed SFV positivity rates of 6% and 5%, respectively [171]. Apart from South Africa and Mozambique already mentioned, SFV is also known from Cameroon, Central African Republic, DR Congo, Nigeria, Senegal and Uganda [164].

#### Sindbis virus (SINV)

Sindbis virus was first isolated in 1952 from a sample of *Cx. pipiens* and *Cx. univittatus* mosquitoes captured in the Sindbis Health District north of Cairo [172]. SINV has since been found to occur widespread within a geographical block running from South Africa up through Africa with known isolations from Uganda, Kenya, Cameroon and Egypt, then through Israel and fanning out deeply into Europe, reflecting migratory bird pathways [115, 173, 174]. There is another north–south block of SINV presence between China and the Philippines down to Australia [173, 174]. In Finland SINV is also known as Pogosta disease and causes regular epidemics involving hundreds or even thousands of human cases, usually in late summer, and similar disease occurs in Sweden (where it is known as Ockelbo disease) and Russia (Karelian fever). SINV was not detected in Finland during seroprevalence surveys for mosquito-borne viruses in humans prior to 1965 [175], then became prevalent and rose considerably between 1981 and 1985, with 0.6% seroprevalence in pregnant women in 1992 [176], rising to 5.2% in a sample of 2529 people tested between 1999 and 2003 in Finland, suggesting that SINV may have been newly introduced into northern Europe sometime during the 1960s to 1970s [177]. In South Africa, during the same outbreak of West Nile virus in 1974 as discussed under WNV above, 16% of the affected human population showed antibodies to SINV [49]. Another dual outbreak of West Nile virus and SINV in 1983 and 1984 in the Witwatersrand - Pretoria area of South Africa resulted in hundreds of Sindbis human cases, but no fatalities. Also in South Africa, tissue samples from 623 horses with unexplained febrile and acute neurologic infections across the country during 2008–2013 showed 1.3% PCR-positive for SINV, 3 of 8 infected horses dying from neurologic sequelea [161]. The disease typically is associated with arthralgia, rash and malaise in humans [106, 110]. In southern Africa SINV cycles within a range of species of wild birds with *Cx. univittatus* mosquitoes as the main vectors in the temperate inland plateau regions, while another bird-feeding species *Culex neavei* is the primary vector in the coastal lowlands of north-eastern South Africa. Both species of mosquitoes are capable of transmitting SINV to humans, although *Cx. univittatus* is a more efficient vector and accounts for the higher prevalence of human cases in the higher-lying inland areas of southern Africa [62]. In Kenya SINV has also been isolated from a range of *Culex* species [93]. There is evidence from genetic studies that migratory birds play a major role in the transcontinental movement of SINV, infecting mainly *Cx. pipiens*, *Cx. torrentium* and *Culiseta morsitans* mosquitoes upon arrival in northern Europe [177]. In Sweden a sero-survey in birds specifically for SINV showed antibody prevalence of 2% in 2002, 8% in 2003, 14% in 2004, and 37% in 2009; highest prevalence was within birds of the family Turdidae [178].

Babanki virus is listed under Togaviridae in Table 1 as some basic data are available, but insufficient for discussion in this section.

### Bunyaviridae

The Bunyaviridae is a large family with medically-important arboviruses clustered into the genera *Orthobunyavirus* (mainly mosquito-borne), *Phlebovirus* (mostly sand fly-borne but some mosquito-borne) and *Nairovirus* (mainly tick-borne). Phylogenetic reconstruction suggests ancestral associations with arthropods at deep nodes indicative of an arthropod origin for all bunyaviruses [179]. Extensive and rapid genetic reassortment within these 3-segmented viruses has resulted in evolutionary proliferation and diversification of virus species, also affecting pathogenicity, host range and vector range [180, 181]. This apparent ease and frequency of genetic reassortment has given rise to occasional uncertainty about assigning some genetically-overlapping viral isolates and could give rise to misunderstandings about disease outbreak descriptions; examples include Batai virus (BATV) and Ngari virus (NRIV) which are almost indistinguishable [41]. The majority of medically-important mosquito-borne viruses in this family occur within the genus *Orthobunyavirus*, most transmitted by *Culex* mosquitoes. *Aedes* also form an important group of vectors and *Anopheles* to a lesser extent. Rift Valley fever with an exceptionally wide range of non-specific mosquito vectors is somewhat of an anomaly among the primarily sandfly-transmitted viruses in the genus *Phlebovirus* [181].

#### Viruses in the genus *Orthobunyavirus* (Bunyaviridae)

##### *Bunyamwera virus(BUNV)*.

Bunyamwera virus was first isolated in 1943 from *Aedes* mosquitoes in the Semliki Forest of Uganda [182] and in 1955 a different strain from *Aedes circumluteolus* mosquitoes in the northern coastal area of KwaZulu-Natal Province in South Africa [183]. Virus was subsequently obtained from the same KwaZulu-Natal Province area from an adult male mosquito catcher presenting with severe headache, neck stiffness and fever [33], while another study in the same area in the 1950s showed 54% seropositivity in adult humans [35]. Serology also indicated BUNV to be common in the Okavango Basin of Botswana and Caprivi Region of Namibia [34]. *Ae. circumluteolus* was commonly found to be infected with the virus and is likely to be important in human-human transmission, even though it is only poorly attracted to people. Neutralizing antibodies were found in a range of water birds in KwaZulu-Natal Province of South Africa [183].

##### *Bwamba virus(BWAV)*.

Bwamba virus was first recovered from febrile road construction workers in the Bwamba Forest (Bundibugyo) region of Uganda in the late 1930s; affected persons typically manifested rapid onset of illness with moderately severe symptoms of fever, headache and backache [31, 32]. Human fatalities have been recorded [28]. BWAV has also been isolated from human patients in multiple other countries in Africa, including Nigeria, Cameroon, Central African Republic, Kenya, Tanzania, Mozambique and South Africa [27]. Despite very low rates of diagnosis and reporting, it is said to be among the most common arthropod-borne diseases in Africa [27], with seropositivity in excess of 90% in some populations [28]. Virus has been isolated from a range of mosquitoes, primarily the anthropophilic *Anopheles funestus* and *An. gambiae* but also *Aedes furcifer* and other *Aedes* species, as well as *Anopheles coustani* and *Mansonia uniformis* [31].

##### *Germistonvirus(GERV)*.

Germiston virus was first isolated in 1958 from mosquitoes collected at a reed-fringed lake in the town of Germiston close to Johannesburg, South Africa. Virus was obtained from a mixed pool of *Culex theileri/Culex rubinotus* and another pool of pure *Cx. rubinotus*. Two laboratory workers became infected while conducting serological studies, one experiencing fever, severe headache and lower back pain, the other with less severe symptoms. Laboratory tests excluded possibility of infection with the closely related Bunyamwera or other viruses [36]. Antibody surveys indicated GERV to be common among residents in the Okavango Basin of Botswana and Caprivi Region of Namibia [34].

##### *Ilesha virus(ILESV)*.

Ilesha virus was recorded for the first time in 1957 from a hospitalized young girl in western Nigeria [37]. The virus has subsequently been isolated from patients in Cameroon, CAR, Nigeria, Senegal, Uganda as well as Madagascar, and antibodies found in persons from Ghana and Niger [184]. Infection with ILESV is accompanied by fever, headache, myalgia of roughly 1 week duration, and in at least two of the very few cases on record led to fatal meningo-encephalitis or fatal haemorrhagic fever [37]. The virus has also been isolated from *Anopheles gambiae* mosquitoes in Central African Republic [185].

##### *Lumbo virus(LUMV)*.

Lumbo virus was isolated from coastal salt-water breeding *Aedes pembaensis* in north-eastern Mozambique in 1959 and 1960, with 100% mortality when inoculated into mice, but primate vervet monkeys, yellow baboon and bush babies developed antibodies without evident illness; 12.5% of a sample of 128 human sera collected in northeastern Mozambique and northern Kwazulu Natal, South Africa, contained neutralizing antibodies [38].

##### *Ngari virus(NRIV)*.

Ngari virus was first isolated from *Aedes simpsoni* mosquitoes in 1979 and from humans in 1993, both in Senegal [186]. The virus was associated with a large-scale outbreak of febrile disease in Sudan in 1988 (in some publications incorrectly ascribed to the closely-related BATV) and in Kenya, Tanzania and Somalia in 1997–1998 [41]; in both sets of outbreaks the events were preceded by unusually heavy rain and flood-promoted mosquito breeding. In the Sudan outbreak at least 18% of the 77,500 febrile cases were clinically diagnosed as malaria, but virus isolates and immunoglobulin from a sample of 195 sera suggest at least 7% were due to NRIV. The large outbreak in Kenya and Somalia involving around 89,000 human infections with 250 deaths was ascribed to Rift Valley fever, but 27% of a sample of approximately 115 acute haemorrhagic cases was due to NRIV based on PCR and/or IgM antibody, while RVF could only be confirmed in 23% of such haemorrhagic cases [41, 43]. NRIV is stated to be more virulent than BUNV and BATV and is associated with hemorrhagic fever [42]. The virus has also been found in sheep and goats in Mauritania [42].

##### *Shuni virus(SHUV)*.

Shuni virus was first isolated in 1966 in Nigeria as part of surveys covering livestock, mosquitoes and *Culicoides* biting midges [48, 164]. It is a cause of neurological disease and associated mortality in domestic and wild animals. Symptoms in horses include depression, anorexia, ataxia, tremors, convulsion, and recumbency with paddling of legs. RT-PCR assays on tissue samples submitted by veterinarians in South Africa indicated 8% SHUV positivity in a sample of 26 horses with fever and 6% of 86 horses with nervous disease [187]. In 2014 two herds of sheep in Israel were noted to show arthrogryposis-hydranencephaly syndrome; 27 tissue samples from these sheep (15 individuals) as well as cattle and goats showed 85% positive for Shuni virus by PCR [188]. SHUV sero-prevalence of 3.9% was found in a sample of 123 South African veterinarians working with horses and wildlife [48]. Shuni virus has been isolated from pools of *Culex theileri* mosquitoes collected near Johannesburg in South Africa [26].

##### *Witwatersrand virus (WITV)*.

Witwatersrand virus was isolated from a pool of 20 *Culex rubinotus* mosquitoes collected at the same lake as Germiston virus was discovered, also in 1958, near the city of Johannesburg, South Africa [53]. WITV was subsequently found to be present fairly regularly in pools of *C. rubinotus* collected in South Africa and Zimbabwe, suggesting that this mosquito is a maintenance vector for WITV and also several other viruses such as Banzi (Flavivirus) and Germiston (Orthobunyavirus) viruses [61]. Nothing is known regarding the significance of WITV for human health, although a serological survey in the north-eastern coastal area of South Africa and in Mozambique suggested the virus to be present in humans in those areas; no antibodies were detected from humans in the Johannesburg Witwatersrand area in which the virus was first discovered. Injection of WITV into infant and adult mice proved lethal [53].

Additional viruses with little published data are listed in Table 1, including Batai, Nyando and Pongola.

#### Viruses in the genus *Phlebovirus* (Bunyaviridae)

##### *Rift Valley fever (RVFV)*.

First described from Kenya in 1931 [189], Rift Valley fever virus (RVFV) has been diagnosed widely across Africa (see Table 1), Madagascar and the Arabian Peninsula [190, 191]. It gives rise to periodic outbreaks sometimes with dramatic impact, such as an outbreak in South Africa in 1950–1951 causing the death of more than 100,000 sheep and 500,000 abortions. Outbreaks also occur amongst other domestic animals such as goats and cattle, major events having occurred in Mozambique, Namibia, Zambia, Zimbabwe and several other southern and eastern African countries [46, 47]. Human infections are regularly associated with such epizootics, including an unprecedented outbreak of up to 200,000 cases and 598 fatalities amongst people in the Nile valley and delta in Egypt in 1977–1978. RVF is mostly recognized from high rates of abortion and high mortality in new-born offspring in domestic ruminants such as sheep, but with associated low numbers of infections and deaths in humans [46, 47]. The disease usually presents itself in humans as mild febrile illness typically with malaise, headache, myalgia, nausea, but can lead to serious complications and death. In a major study to determine vectors of RVFV in Kenya, some 164,626 identified mosquitoes (of 297,000 collected) were sorted in pools of which the following species tested positive, in descending order of frequency: *Aedes mcintoshi/circumluteolus* (26 pools), *Aedes ochraceus* (23 pools), *Mansonia uniformis* (15 pools); *Culex poicilipes*, *Culex bitaeniorhynchus* (3 pools each); *Anopheles squamosus*, *Mansonia africana* (2 pools each); *Culex quinquefasciatus*, *Culex univittatus*, *Aedes pembaensis* (1 pool each) [192]. Various species of *Aedes*, *Culex* and also *Eretmapodites* have also been found positive for RVFV in other studies [26] and also *Mansonia africana* [191]. In southern Africa RVF virus has been isolated from a number of *Aedes* species either from the field or under experimental conditions, the key species most likely to have important roles in virus transmission being *Ae. caballus*, *Ae. juppi*, *Ae. mcintoshi* and *Ae. circumluteolus*. These *Aedes* species probably serve to initiate early-season infection in ruminant hosts which is then expanded into epizootic proportions by more prolific *Culex* species breeding in large bodies of water, such as *Culex theileri*, *Cx. zombaensis* and *Cx. quinquefasciatus* [62, 193]. There is some evidence that RVF virus is carried trans-ovarially to subsequent generations of *Aedes* mosquitoes via eggs that can withstand extended periods of drought [194]. These so-called floodwater breeding *Aedes* species lay their eggs in seasonal pans that dry up during the dry season and the eggs hatch once rain returns the next season or even in subsequent years. Significant effort has been devoted towards developing mathematical and climate models to predict RVF outbreaks [195, 196].

The preceding outline of viruses isolated from Africa, known to infect humans and vectored by mosquitoes, demonstrates not only that several of these viruses have made intercontinental jumps to become major public health challenges, but also that a good number remain in quiescent mainly sylvatic situations with unpredictable potential for future impact, maintaining an “under-the-radar” presence but known to be infective to humans. Table 1 summarizes the discussion above. Below is an outline of the ecological context and other factors that play a role in the potential future spread of one or more of these viruses.

## Predisposing factors favouring the survival, spread and incidence of mosquito-borne arboviruses

Three things are usually necessary for transmission, maintenance and spread of arboviral diseases: the pathogen (virus), the vector (mosquito or tick etc.), and an appropriate virus-replicating host (bird, primate etc.). All three elements must have contact with each other sufficiently often for the virus to make its way along the next link in the cycle to ensure perpetuation of the virus, otherwise local infection and ultimately the species of virus will die out. Sometimes the virus finds alternative pathways for more direct transmission, such as directly between humans through sperm or blood transfusion as with Zika, but these are the exception. Sometimes mosquitoes transmit the virus to a dead-end host, as is the case with many of the arboviruses listed in this paper, where humans are accidental infections that play no essential part in the natural cycling and maintenance of the virus, but it has major consequence because it causes disease or death in humans. The infected humans do not build a sufficiently high viraemia to re-infect other mosquitoes which would enable environmental increase in the virus and therefore increase transmission opportunity and survival chances of the virus; instead the virus is “wasted” in infecting a human which does not lead to onward passage.

However, sometimes an event or situation arises that brings about a quantum jump in the ability of the virus to increase its spread, break out of the historic limiting conditions that restricted its localized distribution and smouldering low-impact presence. These events lead to epidemics and pandemics and major shifts in geographical, economic and health impact. Such break-outs from historic constraints may be temporary or permanent. They include events such as adaptive shifts that enable mosquito populations to depart from a previous sylvatic existence to a strongly human-adapted co-existence, genetic mutations that enable a new strain of virus to more effectively infect and be transmitted by a particular mosquito, mutations that result in entirely new species of viruses that have set up new diseases, and new modes of international transport being provided to mosquitoes pre-adapted to take advantage of such transport. A range of these predisposing factors favouring increase and spread of arboviruses is discussed below.

### Human population growth and its ramifications

The more people there are and the more densely they are spaced, the more hosts and easier accessibility there is for blood-feeding anthropohilic mosquitoes especially in urban environments. Higher human population numbers also mean more waste which creates habitat for container breeders such as *Aedes*, or polluted pools and streams for *Culex*, usually in the extensive poorer and less sanitary city areas with household incomes not allowing adequate personal protection from mosquitoes. It took hundreds of thousands of years for the human population to reach a total of 1 billion people, which it did in the year 1800; it then took 150 years to grow to 2.5 billion people in 1950 [197]. Just short of 70 years later, the global population now stands at over 7 billion people and is projected to reach 8.1 billion by 2025 and 9.6 billion by 2050 [24]. This rapid and massive population growth is associated with increasing urbanization and international migration [197]. In 2014, close on 54% of people lived in urban areas, and projections are that by 2050 about 66% of the global population will reside in cities [198]. Most populations in sub-Saharan Africa are anticipated to double in size [24], translating into increased human movement into historic forested areas to access more land and therefore exposure to the multiple arboviruses existing in sylvatic cycles. It is self-evident that these population increases, increasing urbanization, and increased international traffic will provide very significant opportunity for an increase in spread and growth of mosquitoes and virus and associated arboviral disease.

### Global trade, tourism and other opportunities for geographical leaps

Trans-national and intercontinental transfer of infected vector mosquitoes or their eggs are among the most important methods for rapid geographical leaps in distributions of arboviruses. Examples include transport of *Ae. aegypti* in ships centuries ago to spread yellow fever from Africa to South America, still ongoing since that time [8, 58, 129, 136, 199, 200], and *Ae. albopictus* primarily via the used-car tyre export trade [78, 201–205]. It is precisely this feature of international spread through human-facilitated means [76, 206] that has made dengue, chikungunya, Zika and other viruses such global public health threats and brought *Ae. aegypti* and *Ae. albopictus* such importance as probably the two most important disease transmitters on the planet. Were it not for the international spread of these two mosquitoes by human assistance the arboviruses they transmit would not have anywhere near the impact and importance they currently have, and the diseases would likely still have been confined to their original pockets of historic endemic distribution. West Nile virus too, a *Culex*-associated arbovirus using birds as amplifying host, made the jump to North America [173], apparently from Africa or the Middle East as suggested by genetic evidence [159] and is suspected to have happened via mosquitoes on board aircraft [173].

Aircraft passenger numbers have increased on average nearly 9% per annum in the half-century preceding the mid-2000s [207]. With well over 1 billion foreign arrivals in 2016, international tourism trends have continued to grow. The number of tourists travelling to Asia and the Pacific increased by > 8% in 2016, equalled by > 8% tourism growth into Africa [208]; these are precisely the regions with the greatest reservoirs of arboviruses in the world and projections are that the rises in tourism trends will be sustained [208].

Growth in world trade, despite being referred to as “sluggish”, has shown overall rise in preceding years and is expected to increase by 3.6% in 2017 according to World Trade Organization economists [209]. Shipping traffic increased by more than 27% in the period 1993 to 2006 [207]. The middle class is growing in the emerging economies of Asia, Africa and Latin America, and as disposable income increases, so too does the demand for imported products, directly correlated with shipping [210]. Upon arrival at a new destination, some anthropogenically-relocated vectors - including *Ae. aegypti* - have shown strong competitive ability to displace local mosquito species, enabling them to establish a foothold and disperse [211].

With a steadily expanding human population and a larger middle class with increasing disposable income, international traffic to satisfy tourism and import demands will continue to provide opportunity for movement of vectors and virus, including from Africa where a range of viruses of as yet unknown potential for public health impact are known to occur. Such opportunity for movement of disease is not limited to trade and tourism, as there is a steadily increasing number of people visiting friends and relatives, pilgrimages, humanitarian and other volunteer work, large numbers of politically and ecomically displaced refugees, all contributing to the potential for infected persons to carry pathogens to other geographical shores and infect local vectors [212]. The international or regional movement of livestock, often done informally and *laissez faire* across national boundaries between countries, is a serious risk factor for spread of virus such as Rift Valley fever where local comptetent vectors may be present in as-yet uninfected countries [213].

### Vertical trans-generational transmission of virus

Transmission of virus between mosquitoes, birds, humans and other vertebrates is known as horizontal transmission, but there is also trans-generational vertical transmission of virus within some vector species allowing passage of infective virus from adult mosquitoes to offspring [214]. The terms “transovarial transmission” and “vertical transmission” are sometimes used interchangeably by some authors, despite transovarial passage being a subset of vertical transmission. Such vertical transmission of virus from one generation of mosquitoes to the next appears uncommon among mosquito-borne alphaviruses, has intermediate occurrence in flaviviruses but high frequency among bunyaviruses [215]. Alphaviruses have been found on the egg-surfaces but the egg rarely becomes infected, apparently due to absence of virus in the ovarioles themselves but presence in the lateral and common oviducts where contamination could occur. A similar situation exists in several flaviviruses that have been studied, where eggs become infected by a poorly understood mechanism, which is also not transovarial as the follicles are not involved in infection and instead the mature egg is somehow infected during oviposition. True transovarial transmission happens among several bunyaviruses, by way of germinal tissue infection. The latter method results in very high vertical transmission rates [215].

Among flaviviruses, vertical transmission of dengue-2 virus (DENV-2) in *Ae. albopictus* has been demonstrated in laboratory studies in India [216]. Angel and Joshi [217] collected larvae from pots and other containers in domestic and peri-domestic areas in Jodhpur, Jaipur and Kota districts in India, reporting vertically transmitted dengue virus in up to 13.3% of *Ae. aegypti*, 18.7% of *Ae. albopictus*, and and 20% *of Ae. vittatus* adults reared from such collected larvae. Natural vertical transmission of dengue virus has also been demonstrated in north-eastern Brazil, the authors reporting DENV-2 and DENV-3 from *Ae. albopictus*, and DENV-2 from *Ae. aegypti*, these mosquitoes having been reared from larvae and pupae collected in households in Fortaleza city [218]. Experimental transovarial and vertical transmission of YFV has also been confirmed for *Ae. aegypti* in several studies [219–221], while natural vertical transmission of YFV by *Ae. aegypti* was confirmed in Senegal, Africa [222]. Such vertical transmission is not limited to *Aedes* however. Experimental and natural vertical transmission of WNV has been reported for *Culex tarsalis* and *Culex pipiens* complex in California, including multiple examples of female *Culex* passing WNV to progeny [223]. Experimental vertical transmission of WNV was also demonstrated in *Culex tritaeniorhynchus*, and also in *Ae. aegypti* and *Ae. albopictus* [224]. *Haemagogus equinus* females were also experimentally infected in Panama with DENV-1 and this was successfully passed to egg-stage and subsequently to fourth-stage larvae [225].

Among the alphaviruses, vertical transmission of CHIKV has been confirmed in *Aedes aegypti* mosquitoes in northern India [226], and in Thailand it was vertically transmitted experimentally to F5 and F6 generations of *Ae. aegypti* and *Ae. albopictus*, respectively, with *Ae. albopictus* being more susceptible and with greater ability for vertical transmission [227].

At least one publication suggests that trans-generational vertical transmission of virus, at least in the case of dengue, is unlikely to be important in the epidemiological persistence of dengue viruses [228].

### Dessication-resistant eggs

The ability to pass virus on from one mosquito generation to the next by way of infected eggs (vertical transmission) is a major adaptive advantage to increase the likelihood of virus survival in the environment, helping to reduce the chances of local extinction of the virus at a particular geographical focus. However, the combination of this trait - successful passage of viable virus from one female mosquito via the eggs and larvae to the next generation of adult females - with eggs that are also desiccation resistant adds a truly powerful dual mechanism not only for survival of virus but also geographical spread. This phenomenon of some mosquitoes being able to lay eggs that can survive variable periods of complete drying out and hatch at the next inundation by rain or floods or humans watering a garden, and even have staggered hatching events spread over multiple inundations, is probably the single most important biological trait that (in combination with strong anthropophily) has allowed such effective global spread of *Ae. aegypti* and *Ae. albopictus* and as a consequence also their associated diseases of DENV, CHIKV, YFV and others.

The phenomenon of being able to lay drought - or desiccation-resistant eggs is best known within the *Aedes* subgenus *Stegomyia*, species of which tend to be container breeders laying eggs in small volumes of water subject to drying up, hence placing an evolutionary advantage on populations which developed the ability to survive such episodes of drying up. These species now even lay their eggs above the water-line in small pools, effectively encouraging drying out and survival until the next inundation or flooding event when more water is likely to be available for the full larval development period [229].

The ability to lay desiccation-resistant eggs has been proven for multiple *Aedes* (*Stegomyia*) species, including *Ae. aegypti* and *Ae. albopictus* [229–231]. *Aedes aegypti* eggs have been shown to survive under very different humidity regimes, with a mean dry-egg survival period of 128 days at 88% relative humidity, while *Ae. albopictus* generally had lower survival with mean of 78 days at 88% RH [229]. Other studies have confirmed survival of *Ae. aegypti* dry eggs up to or exceeding 4 months in Tanzania [232, 233] or 5 months in Queensland, Australia [234] under outdoor natural conditions; reports also exist for egg survival of *Ae. albopictus* exceeding 5 months [235]. It is therefore clear that both *Ae. aegypti* and *Ae. albopictus* could easily survive extended periods of trans-oceanic transport as dry eggs in car-tyres or other containers.

One of the enduring puzzles has been how RFV is maintained between epidemics which may have a decade or more separating such events; speculation has long existed that transovarially-infected or vertically transmitted eggs of *Aedes* that are drought-resistant and survive for many years may be responsible or contribute to such inter-epidemic survival [194, 196].

### Virus mutations

Virus evolution by way of mutation, genome segment reassortment or recombination is one of the contributory factors to the occurrence of emergent and/or re-emergent viral diseases [236]. High frequency reassortment can occur when mosquitoes are infected with two viruses most likely arising from interrupted feeding and moving from one host to another to complete a blood-meal, or separate infected bloodmeals one or 2 days apart. That such reassortment does happen is well established, for example Ngari virus which is a reassortment between Bunyamwera and Batai virus [43, 236]. Reassortments of different strains of Rift Valley fever virus have also been found in East Africa [237]. The single-stranded RNA bunyaviruses are particularly prone to produce genetic variants and many of these variants have been detected from field situations [238]. Not only are reassortments able to produce new viruses with new epidemiologies, virulence and pathologies, but relatively minor mutations are also capable of dramatically affecting vector susceptibility enabling quantum improvements in transmission efficiency in previously inefficient vectors or even new vectors to transmit the virus. A case in point is the single amino acid change in the EI envelope glycoprotein of chikungunya virus. CHIKV historically had an alanine amino acid at position 226 in EI and this virus was transmitted mainly by *Ae. aegypti*; a single mutation resulting in a valine amino acid at position 226 greatly enhanced virus replication within - and transmission by - *Ae. albopictus* [153, 239, 240] and led to the widespread dispersal of CHIKV in regions of Asia where *Ae. albopictus* predominated [236], as well as an outbreak in northern Italy where *Ae. aegypti* did not occur [241]. These events all potentially contribute to the survival, spread and increased incidence of mosquito-borne arboviruses.

### Vector mutations and adaptations

Not only do the viruses that cause disease mutate or show adaptive selection for optimized transmission, but so do the vectors. *Aedes aegypti* is one example. This historically forest-dwelling zoophilic species adaptively transformed into a highly anthropophilic urban-adapted “domesticated” form responsible for transmitting several of the most serious public health challenges on the planet. The “domestication” adaptation enabled *Ae. aegypti aegypti* to spread with humans to almost all tropical and sub-tropical parts of the world, where it is now a major vector for dengue, chikungunya, and yellow fever in some regions [242, 243]. Other examples also exist of adative shifts that enable vector mosquitoes to evade control attempts, resulting in dramatic resurgence in disease. Such examples are common especially in malaria control, where vector resistance to pyrethroid and other insecticides has evolved on numerous occasions in multiple countries, causing serious setbacks in disease control programmes [244–249]. Similar and escalating trends of insecticide resistance have also been recorded for non-anopheline species, such as *Ae. aegypti* [250–254], to some extent in *Ae. albopictus* [251, 254, 255] and various *Culex* species including *Cx. pipiens* [256] and *Cx. quinquefasciatus* [256–258].

### Climate change

Climate change embraces the suite of projected climatic parameters that are already in the process of shifting from historic norms and “natural” background long-term cycles, these changes brought about by massive anthropogenic emmissions of gases that affect meteorological processes. The expert Intergovernmental Panel on Climate Change (IPCC) produces periodic assessment reports which consistently show a steady warming of the planet that is set to continue for the remainder of this century [259]. The consequences of these “greenhouse-effect” rising temperatures will be fundamental and wide-ranging changes in average daily temperatures, precipitation, flood and drought events and various other climate-related parameters, but with a broad likelihood that these changes will in many geographical regions favour the spread of disease vectors and their abundance [239, 260]. However, the interaction of various factors is complex, making predictions of likely vector increases or disease spread difficult [236, 261].

Global warming and other climate change-related factors have already resulted in predictions of further spread of *Ae. albopictus* in Europe by 2030 [236, 261, 262]. The spread and establishment of arboviruses such as RVFV in Europe and the USA has also been discussed in relation to climate change [239].

## Conclusions

The literature reviewed in this paper shows that there is a substantial number of mosquito-borne arboviruses present in Africa, and that several of these have already in preceding centuries or decades escaped from their historic African areas of origin to become serious public health threats at global scale. Zika is currently the most recent example of this. Many of these mosquito-borne arboviruses remain quiescent but almost certainly some of them must surely have the potential to break out under “Perfect Storm” conditions to invade other geographical regions with unknown consequences. Variour factors increase this likelihood, including expanding human populations that of necessity clear forests and woodland for cropland and increased needs of sustenance, thereby placing themselves within reach of sylvatic disease cycles, plus the ever-increasing international travel and ease with which infected vectors or people can carry virus to every inhabited corner of the globe. It is therefore incumbent upon nations and governments to maintain and indeed increase vigilance against the insidious infiltration of vectors and viruses. All it takes is for a few infected mosquitoes or people or animals to establish the vector or virus which slowly simmers and grows until it emerges as a full-blown outbreak and public health emergency. This is the historic pattern of most of these diseases, be it yellow fever, dengue, chikungunya, West Nile virus, Zika and several others. Africa appears to be not only the ancestral cradle of humankind, but also the spawning ground of many zoonotic diseases, especially arboviruses. It is very likely that Africa has the greatest potential for novel zoonoses and for the next export of a previously quiescent pathogen to invade elsewhere on a rapidly changing planet.

## Abbreviations

*Ae*: *Aedes*
AMTV: Arumowot virus
*An*: *Anopheles*
BAGV: Bagaza virus
BANV: Banzi virus
BATV: Batai virus
BBKV: Babanki virus
BOUV: Bouboui virus
BUNV: Bunyamwera virus
BWAV: Bwambwa virus
CHIKV: Chikungunya virus
*Cx*: *Culex*
DENV1–4: Dengue virus serotypes 1 to 4
GERV: Germiston virus
ILEV: Ilesha virus
KEDV: Kedougou virus
LUMV: Lumbo virus
MIDV: Middelburg virus
MOSV: Mossuril virus
NDUV: Ndumu virus
NDV: Nyando virus
NRIV: Ngari virus
NTAV: Ntaya virus
ONNV: O’nyong-nyong virus
PGAV: Pongola virus
RVFV: Rift Valley fever virus
SFV: Semliki Forest virus
SHUV: Shuni virus
SINV: Sindbis virus
SPOV: Spondweni virus
UGSV: Uganda S virus
USUV: Usutu virus
VEE: Venezuelan equine encephalitis virus
WESV: Wesselsbron virus
WITV: Witwatersrand virus
WNV: West Nile virus
YAOV: Yaounde virus
YFV: Yellow fever virus
ZIKV: Zika virus
